# Phloretin Inhibits Quorum Sensing and Biofilm Formation in *Serratia marcescens*

**DOI:** 10.3390/molecules28248067

**Published:** 2023-12-13

**Authors:** Yueheng Qi, Pengcheng Ji, Kunyuan Yin, Yi Zheng, Jiangxiu Niu, Aiqun Jia, Jinwei Zhou, Jingguo Li

**Affiliations:** 1 Henan Provincial People’s Hospital, People’s Hospital of Zhengzhou University, Zhengzhou 477150, China; 2 Luoyang Key Laboratory of Organic Functional Molecules, College of Food and Drug, Luoyang Normal University, Luoyang 471934, China; 3 School of Food and Biological Engineering, Xuzhou University of Technology, Xuzhou 221018, China

**Keywords:** *Serratia marcescens*, phloretin, quorum sensing, biofilm, virulence

## Abstract

This study investigated the antivirulence capacity and mechanism of apple-skin-derived phloretin against *Serratia marcescens* NJ01, a vegetable spoilage bacterium. At 0.5 to 2 mg/mL doses, phloretin considerably inhibited the secretion of acyl homoserine lactones (AHLs), indicating that phloretin disrupted quorum sensing (QS) in *S. marcescens* NJ01. The dysfunction of QS resulted in reduced biofilms and the decreased production of protease, prodigiosin, extracellular polysaccharides (EPSs), and swimming and swarming motilities. Dysfunctional QS also weakened the activity of antioxidant enzymes and improved oxidative injury. The improved oxidative injury changed the composition of the membrane, improved membrane permeability, and eventually increased the susceptibility of biofilm cells to amikacin, netilmicin, and imipenem. The disrupted QS and enhanced oxidative stress also caused disorders of amino acid metabolism, energy metabolism, and nucleic acid metabolism, and ultimately attenuated the ability of *S. marcescens* NJ01 to induce spoilage. Our results indicated that phloretin can act as a potent drug to defend against spoilage by *S. marcescens*.

## 1. Introduction

Food spoilage caused by foodborne pathogens has attracted global attention due to its resulting economic loss and foodborne illness [1]. *Serratia marcescens* is a Gram-negative bacterium that is widely found in soil, air, vegetables, meat, and dairy products [2,3]. *S. marcescens* is responsible for the vegetable yellow vine disease that induces leaf yellowing, withering, and even death [4]. Chemical drugs are adopted as the main measures for preventing and treating vegetable disease [5]. However, the extensive application of chemical drugs has led to serious drug resistance [5], and drug residues in food products also pose a threat to the environment and human health [6]. Therefore, new approaches to defend against infections induced by *S. marcescens* without extensive fungicidal use are urgently needed.

The formation of biofilms is among the most important reasons why *S. marcescens* attains drug resistance [7]. Biofilms are bacterial communities, in which cells are surrounded by a dense matrix consisting of proteins, lipids, EPS, and nucleic acids that can prevent drugs from entering cells [8]. Studies have demonstrated that biofilm formation of *S. marcescens* is controlled by quorum sensing (QS), a contact medium utilized by bacteria to regulate their colony action [9]. Acyl homoserine lactones (AHLs) are the main signaling molecules secreted by *S. marcescens* [3]. *S. marcescens* secretes C4-C8 AHLs to mediate biofilm development, infection, drug resistance, and the secretion of virulence factors [3]. Therefore, disrupting the QS of *S. marcescens* is a compelling technique to diminish the drug resistance and virulence of *S. marcescens*.

The high toxicity of chemically synthesized drugs limits their widespread application in the food industry [10]. Natural compounds derived from medicinal and edible plants have received great attention due to their high inhibitory activity against QS and low toxicity [11]. For instance, hordenine derived from sprouting barley showed potent anti-QS activity against the spoilage bacterium *S. marcescens* [3]. Petroselinic acid isolated from many plant seed oils could significantly inhibit the biofilms and QS of *S. marcescens* [12]. Phytol in Piper betle considerably reduced the virulence and pathogenicity of *S. marcescens* by interrupting the QS system of *S. marcescens* [13]. Phloretin, a phenolic compound, is abundant in the peel and root bark of juicy fruits, such as apples and pears. Previous studies have shown that phloretin could inhibit the virulence and biofilm formation of *Listeria monocytogenes* and *Pectobacterium brasiliense* by affecting their QS systems [14,15]. However, whether phloretin can decrease the virulence of *S. marcescens* has yet to be documented. Here, the QS and biofilm inhibitory potential of phloretin against *S. marcescens* NJ01 was evaluated, and the synergistic mechanism by which phloretin improves the susceptibility of biofilms to antibiotics was clarified.

## 2. Results

### 2.1. Growth Curve

**Figure 1:**
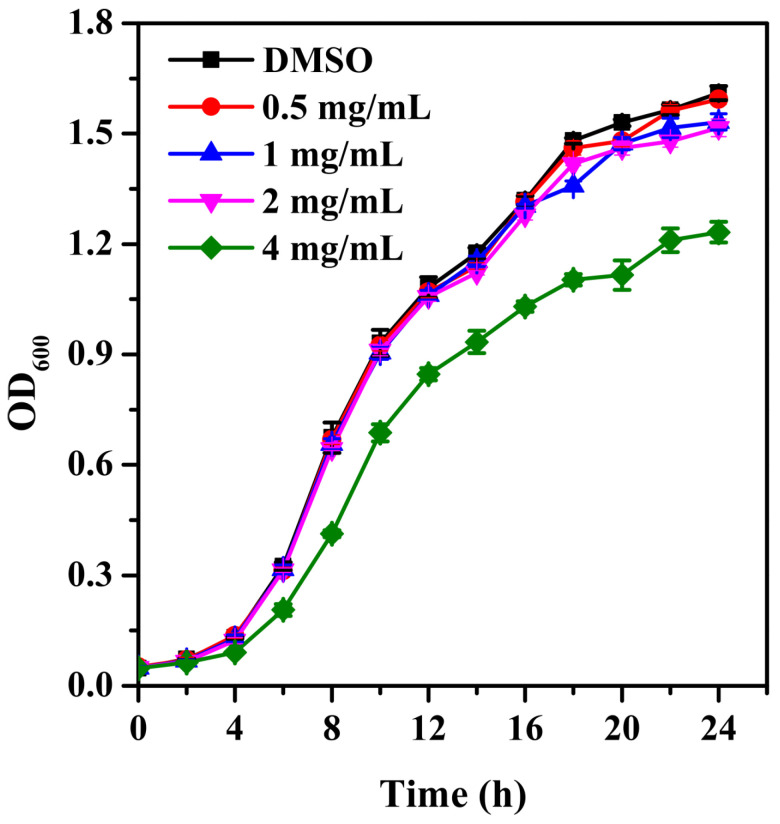
Growth profile of *S. marcescens* NJ01 treated with 0.5, 1, 2, and 4 mg/mL of phloretin for 24 h.

As presented in Figure 1, phloretin at a concentration of 4 mg/mL has a side effect on the cell growth of *S. marcescens* NJ01. When the concentration of phloretin is lower than 2 mg/mL, it had no antimicrobial effect on *S. marcescens* NJ01 (Figure 1).

### 2.2. AHL Production

**Figure 2:**
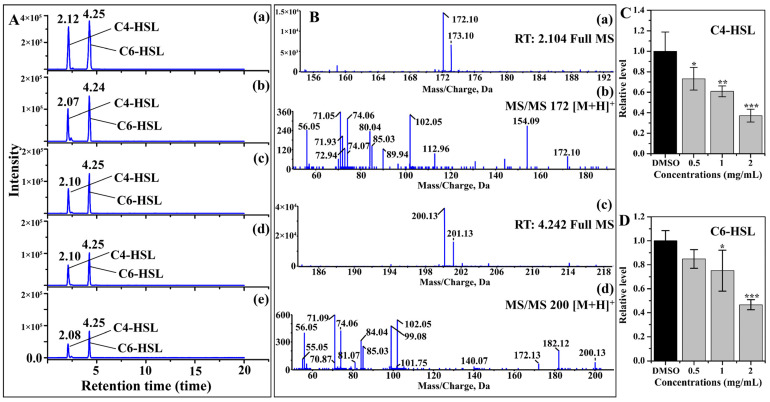
Effect of phloretin on AHL secretion. (**A**) HPLC chromatograms of C4-HSL and C6-HSL exposure to (b) DMSO, (c) 0.5, (d) 1, and (e) 2 mg/mL of phloretin. (a) Standard chemicals of C4-HSL and C6-HSL. (**B**) MS and MS^2^ spectra of C4-HSL and C6-HSL, respectively: (a) and (c) represented full MS spectra of C4-HSL and C6-HSL, respectively; (b) and (d) represented MS^2^ spectra of C4-HSL and C6-HSL, respectively. (**C**) and (**D**) represented quantitative analysis of C4-HSL and C6-HSL, respectively. *, *p* < 0.05 versus DMSO control. **, *p* < 0.01 versus DMSO control. ***, *p* < 0.001 versus DMSO control.

The AHLs of C4-HSL and C6-HSL were detected based on the peak time and their MS and MS^2^ ions (Figure 2A,B). A relative quantification analysis indicated that the secretion of C4-HSL and C6-HSL was significantly reduced after treatment with phloretin (Figure 2A,C,D). Exposure to 0.5, 1, and 2 mg/mL phloretin diminished C4-HSL levels by approximately 30%, 40%, and 60%, respectively (Figure 2C). After 1 and 2 mg/mL phloretin was applied, the production of C6-HSL decreased to 75% and 46%, respectively (Figure 2D). Therefore, the QS of NJ01 was gravely disrupted by phloretin exposure.

### 2.3. Biofilm Formation

**Figure 3:**
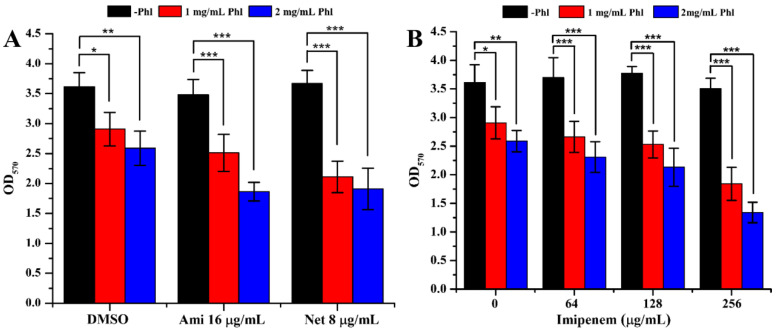
The synergistic effect of phloretin (Phl) and amikacin (Ami) (**A**), netilmicin (Net) (**A**), and imipenem (**B**) on biofilm formation of *S. marcescens* NJ01. (*) *p* < 0.05; (**) *p* < 0.01; (***) *p* < 0.001 versus the DMSO control.

As presented in Figure 3, the biofilm biomass was decreased by approximately 20% and 28% after exposure to 1 and 2 mg/mL phloretin, respectively. The antibiotics amikacin, netilmicin, and imipenem used individually exhibited no inhibitory effect on biofilm formation. However, when exposed to these antibiotics alongside phloretin, the inhibitory effect was remarkably improved. Exposure to 16 µg/mL amikacin and 8 µg/mL netilmicin alongside 2 mg/mL phloretin reduced biofilm formation by approximately 50% (Figure 3A). After exposure to 256 µg/mL imipenem and 2 mg/mL phloretin, the inhibitory impact was enhanced to 63% (Figure 3B).

**Figure 4:**
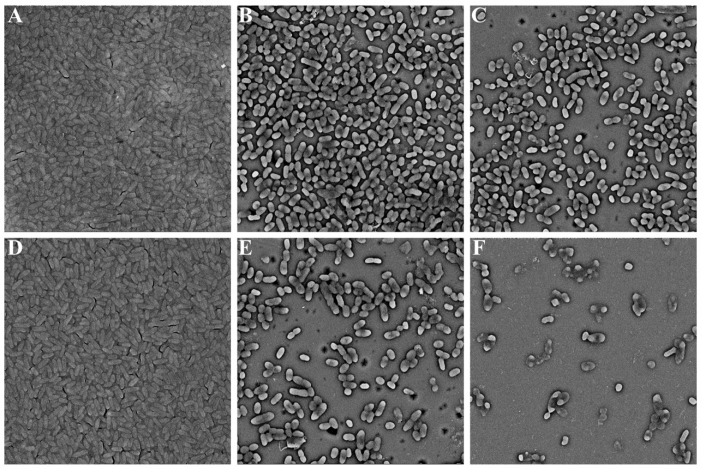
SEM (8μm) analysis of biofilms exposure to (**A**) DMSO, (**B**) 1 mg/mL of phloretin, (**C**) 2 mg/mL of phloretin, (**D**) 16 µg/mL of amikacin, (**E**) 1 mg/mL of phloretin + 16 µg/mL of amikacin, and (**F**) 2 mg/mL of phloretin + 16 µg/mL of amikacin, respectively.

The SEM images presented that the untreated biofilms exhibited a thick and three-dimensional structure adhered with extracellular polymers (Figure 4A). However, after exposure to 1 and 2 mg/mL phloretin, the cells were separated and the attached extracellular polymers were significantly decreased (Figure 4B,C). Treatment with 16 µg/mL amikacin individually showed no obvious inhibition of biofilm formation in *S. marcescens* NJ01 (Figure 4D). However, after exposure to amikacin in combination with phloretin, a significant improvement in the reduction of biofilms was observed (Figure 4E,F). The remaining cells were well separated and the integrity of some bacterial cells was seriously disrupted (Figure 4).

### 2.4. Inhibition of Virulence Factors

**Figure 5:**
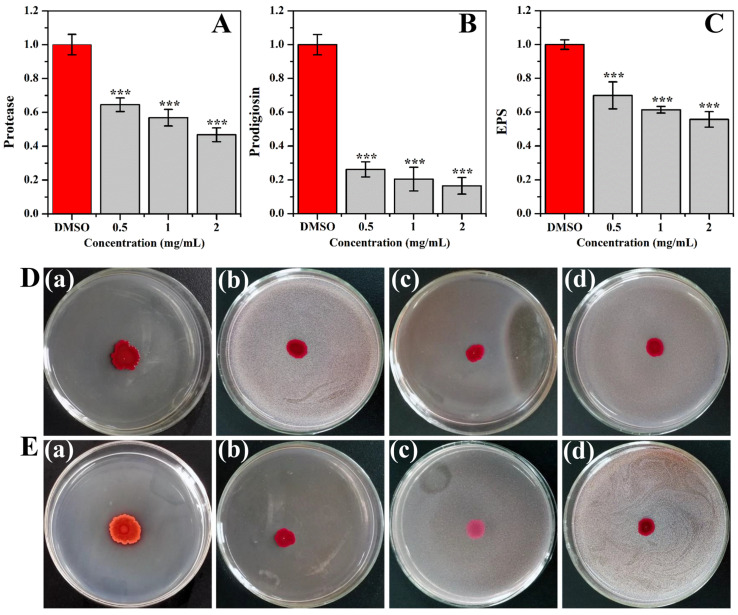
Impact of phloretin on virulence factors of (**A**) protease activity, (**B**) prodigiosin production, (**C**) EPS production, and (**D**) swimming and (**E**) swarming motility. (a), (b), (c), and (d) represented treatment with DMSO, 0.5, 1, and 2 mg/mL of phloretin, respectively. (***) *p* < 0.001 versus the DMSO control.

Phloretin exposure at 1 and 2 mg/mL inhibited protease activity by 43% and 53%, respectively (Figure 5A). The inhibitory effect of phloretin on prodigiosin production is presented in Figure 5B. The results indicated that phloretin administration at 0.5, 1, and 2 mg/mL resulted in a reduction of prodigiosin by more than 73% (Figure 5B). The EPS quantification indicated that the EPS production was significantly inhibited after exposure to phloretin (Figure 5C). Phloretin exposure at 0.5, 1, and 2 mg/mL decreased EPS production by approximately 30%, 39%, and 44%, respectively. Furthermore, the swimming and swarming motilities were also considerably repressed with phloretin administration (Figure 5D,E).

### 2.5. Metabolic Analysis

**Figure 6:**
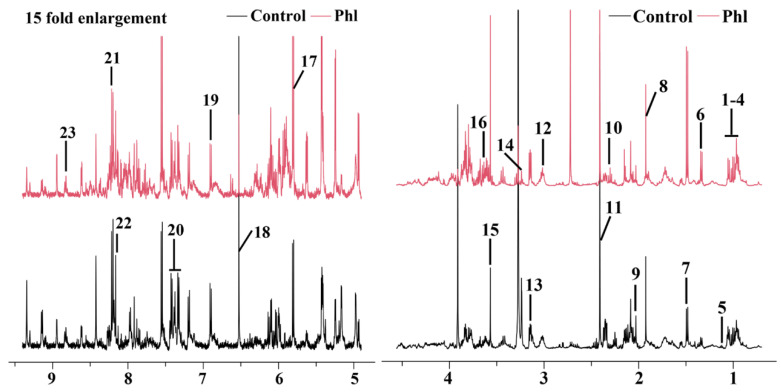
^1^H NMR spectra of *S. marcescens* NJ01 extracts from phloretin-treated (red line) and control groups (black line). Labeled metabolites: 1, glycocholate; 2, isoleucine; 3, leucine; 4, valine; 5, 3-methyl-2-oxovalerate; 6, lactate; 7, alanine; 8, acetate; 9, 2-aminoadipate; 10, glutamate; 11, succinate; 12, 2-oxoglutarate; 13, ethanolamine; 14, betaine; 15, glycine; 16, maltose; 17, uracil; 18, fumarate; 19, tyrosine; 20, phenylalanine; 21, hypoxanthine; 22, NAD^+^; 23, nicotinate.

**Figure 7:**
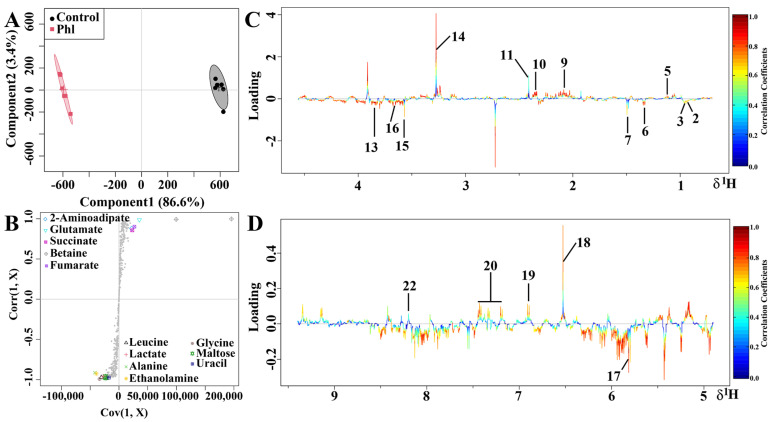
OSC−PLS−DA of metabolomics profiles from phloretin-treated and control groups. (**A**) Score plot. Component 1 and component 2 explained 90% of total variance in the extracts of *S. marcescens* NJ01; (**B**) S-plot points represent different variables (metabolites); (**C**,**D**) Color-coded loading plot after removal of water signals and affected regions. Color bars in red and blue represent metabolites that significantly or indistinctively contributed to the separation of groups, respectively. Peaks in positive and negative status indicate decreased and increased metabolites relative to the score plot in the phloretin-treated group.Labeled metabolites: 1, glycocholate; 2, isoleucine; 3, leucine; 4, valine; 5, 3-methyl-2-oxovalerate; 6, lactate; 7, alanine; 8, acetate; 9, 2-aminoadipate; 10, glutamate; 11, succinate; 12, 2-oxoglutarate; 13, ethanolamine; 14, betaine; 15, glycine; 16, maltose; 17, uracil; 18, fumarate; 19, tyrosine; 20, phenylalanine; 21, hypoxanthine; 22, NAD+; 23, nicotinate.

The typical 500 MHz ^1^H NMR spectra of *S. marcescens* NJ01 extracts obtained from the DMSO- and phloretin-treated groups are shown in Figure 6. Assignments of metabolites were based on chemical shifts and by querying publicly accessible metabolomics databases such as the Kyoto Encyclopedia of Genes and Genomes (KEGG, http://www.kegg.jp, accessed on 23 October 2023) and the Human Metabolome Database (HMDB, http://www.hmdb.ca, accessed on 23 October 2023). A total of 23 metabolites were assigned. The assigned compounds mainly corresponded to amino acids, organic acids, nucleotides, amines, and metabolites involved in the energy supply. The detailed compounds and their identification, chemical shifts, and fold changes are shown in Table 1. The OSC-PLS-DA plot showed that the DMSO- and phloretin-treated groups exhibited notable discrimination (Figure 7A), indicating that significant metabolic changes occurred in these two groups. The vital variables in the S-plot exhibited various colors and shapes, and contributions of those metabolites to the grouping were related to their distance to the center: variables that were further from the center gained more significant contributions to the group separation (Figure 7B). On the basis of correlation coefficients, the loading plots (Figure 7C,D) were coded with a cool color and warm color tone; from blue to red, the relativity gradually enhanced. The metabolites isoleucine, leucine, lactate, alanine, ethanolamine, glycine, maltose, and uracil were notably increased, while 3-methyl-2-oxovalerate, 2-aminoadipate, glutamate, succinate, betaine, fumarate, tyrosine, phenylalanine, and NAD^+^ were notably decreased after exposure to phloretin (Figure 7C,D).

### 2.6. Oxidative Damage and Membrane Permeability

**Figure 8:**
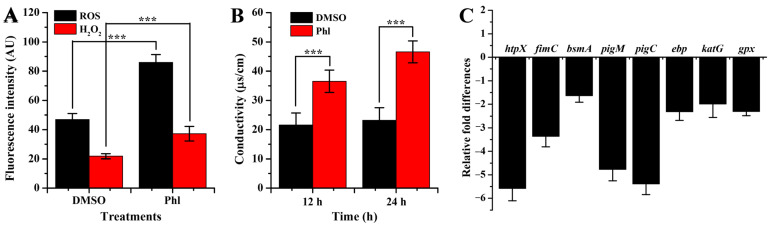
Oxidative stress (**A**), membrane permeability (**B**), and gene expressions (**C**) induced by phloretin. (***) *p* < 0.001 versus the DMSO control.

The levels of ROS and H_2_O_2_ were notably improved after exposure to 2 mg/mL of phloretin (Figure 8A). The results implied that the bacterial cells of NJ01 underwent serious oxidative stress after phloretin exposure. Furthermore, the permeability of the cell membrane was also investigated. The results indicated that membrane permeability was notably improved after phloretin administration (Figure 8B). This improvement was intensified as incubation continued.

### 2.7. RT-qPCR Analysis

The expressions of the genes *htpX*, *fimC*, *bsmA*, *pigM*, *pigC*, *ebp*, *katG*, and *gpx*, which are responsible for protease, fimbriae, adherence, prodigiosin synthesis, extracellular polysaccharides, catalase, and glutathione peroxidase, respectively, were downregulated by 5.6-, 3.7-, 1.6-, 4.8-, 5.4-, 2.3-, 2.0-, and 2.3-fold, respectively, after exposure to phloretin (Figure 8C). These genes were correlated well with virulence factor secretion, biofilm formation, antibiotic resistance, and oxidative injury. This implied that the virulence of NJ01 may be attenuated by phloretin exposure.

## 3. Materials and Methods

### 3.1. Bacterial Growth

*S. marcescens* NJ01, one spoilage bacterium obtained from rotten tomatoes, was incubated in Luria Bertani (LB) medium at 28 °C [3]. The phenolic compound phloretin was isolated from apple skin and dissolved in dimethyl sulfoxide (DMSO). The minimum inhibitory concentration (MIC) of phloretin against NJ01 was measured through a two-fold dilution procedure [16]. To determine the growth profile, overnight cultures of NJ01 (OD600 = 0.5) were 0.1% inoculated into LB broth with varying concentrations of phloretin (0.5–4 mg/mL). DMSO were used as the negative control. Bacterial growth was determined using a microplate reader at 600 nm (Synergy H1, BioTek, Winooski, VT, USA).

### 3.2. Inhibition of AHL Production

Overnight cultures of NJ01 (OD_600_ = 0.5) were 0.1% (*v*/*v*) inoculated into LB broth with varying concentrations of phloretin (0.5–2 mg/mL). After 24 h cultivation, the mixtures were centrifuged and extracted with acidified ethyl acetate (1:1, *v*/*v*). The organic solvent was evaporated under reduced pressure and the residues were redissolved in methanol, and high-performance liquid chromatography–mass spectrum (HPLC-MS) was employed for AHL analysis. The AHLs were detected according to the MS^2^ fragment ions of standard C4-HSL and C6-HSL and their retention time. The AHL production was normalized to the DMSO-treated samples as described previously [17].

### 3.3. Biofilm Development

Overnight cultures of NJ01 (OD_600_ = 0.5) were 0.1% (*v*/*v*) inoculated into trypticase Soytone broth (TSB) with different concentrations of phloretin combined with or without 16 µg/mL amikacin, 8 µg/mL netilmicin, and 64, 128, and 256 µg/mL imipenem, respectively. The cultures were incubated at 28 °C for 24 h without shaking and then washed using phosphate buffer saline (PBS) to remove planktonic cells. The formed biofilms were stained with crystal violet, dissolved in ethanol, and then determined by reading the optical density (OD) value at 570 nm [18].

### 3.4. Microscopic Analysis

Overnight cultures of NJ01 (OD_600_ = 0.5) were 0.1% (*v*/*v*) inoculated into TSB medium with different concentrations of phloretin and 16 µg/mL amikacin in 24-well chambered cover slides. The cultures were cultivated at 28 °C for 24 h without shaking. The mature cells were washed with distilled water, fixed with 2.5% glutaraldehyde, and then dehydrated using graded ethanol. Subsequently, biofilms were freeze-dried, coated with gold, and analyzed using scanning electron microscopy (SEM) (JSM6360, JEOL, Tokyo, Japan) [7,19,20].

### 3.5. Virulence Factors

Bacterial seed solution of NJ01 (0.1%, *v*/*v*) was inoculated into LB broth with varying concentrations of phloretin (0.5–2 mg/mL). The 24 h cultures were centrifuged at 4 °C for 10 min. The supernatant was mixed with 0.3% azocasein (Sangon Biotech, Shanghai, China) in a volume ratio of 5:3, and 1.2 mL of 1 g/dL trichloroacetic acid was supplemented to precipitate the undigested substrate for 20 min. After centrifugation, the supernatant was added to 1.2 mL of 1 mol/L NaOH to stop the reaction. The protease activity was evaluated by recording the OD_440_ value [21].

For prodigiosin secretion, 1 mL of the cultures were centrifuged at 4 °C for 10 min and the obtained cells for pellets were extracted with 1 mL acidified ethanol (4%, 1 M HCl). Prodigiosin was determined by measuring OD_534_ [21].

For the extracellular polysaccharide (EPS) assay, the formed biofilms on the coverslips were washed with PBS and then added with NaCl–phenol–hydrazine sulfate mixture [21]. The mixture was incubated without light for 60 min, and EPS production was assessed by recording OD_490_ [21].

Swimming and swarming assays were performed by inoculating 1 μL *S. marcescens* NJ01 cultures into the swimming (1% tryptone, 0.5% NaCl, 0.3% agar, pH 7.2) and swarming medium (1% tryptone, 0.5% NaCl, 0.5% glucose, 0.3% agar, pH 7.2) as described previously. Bacterial cells were incubated at 28 °C for 24 h, and the movement status was recorded [21,22].

### 3.6. Metabolomics Analysis

Bacterial seed solution of NJ01 (0.1%, *v*/*v*) was inoculated into LB broth with 2 mg/mL phloretin. After 24 h of incubation, bacterial cells were gathered through 10 min of centrifugation at 10,000 rpm. Bacterial cells were washed with PBS and then extracted with methanol/water/chloroform mixture in a volume ratio of 10:9:20. The mixture was centrifuged at 4 °C at 10,000 rpm for 10 min, and the upper layer was collected for lyophilization. The dried samples were redissolved in D_2_O phosphate buffer and then transferred to nuclear magnetic resonance (NMR) tubes for NMR analysis [23,24].

### 3.7. Analysis of Reactive Oxygen Species (ROS) and H_2_O_2_

ROS were evaluated by adding 6-carboxy-2′,7′-dichlorodihydrofluorescein diacetate to NJ01 cells. After incubation for 30 min, the mixture was centrifuged and bacterial cells were collected. The obtained cells were resuspended in PBS and analyzed at 485 nm for excitation and 525 nm for emission [25]. To measure H_2_O_2_, the obtained cells were resuspended in PBS. H_2_O_2_ was released from the cells and equilibrated in the solutions. The mixture was centrifuged and the supernatant was used for H_2_O_2_ analysis using the horseradish peroxidase–scopoletin method as described by Gonzalez-Flecha and Demple (1997) [26].

### 3.8. Membrane Permeability

The cultures of NJ01 were centrifuged and bacterial cells were obtained. Bacterial cells were washed with 5% glucose until their electric conductivities were near to that of the 5% glucose, and then the strains became isotonic strains. The 5% glucose was supplemented with 2 mg/mL of phloretin and their electric conductivities were determined as *L*_1_. The isotonic strain solution was supplemented with 2 mg/mL of phloretin and incubated at 37 °C for 8 h. After incubation, the electric conductivities were determined as *L*_2_. The conductivity of bacteria in 5% glucose treated in boiling water for 5 min was determined as *L*_0_. The cell membrane permeability was determined as described by Diao et al. (2014) using the formula [27]:

the relative electric permeability (%) = 100 × (*L*_2_ − *L*_1_)/*L*_0_.

### 3.9. Gene Expression

*S. marcescens* NJ01was grown in LB medium with or without 2 mg/m phloretin at 28 °C at 180 rpm for 24 h. After incubation, cells were washed with sterile PBS and collected after 10 min centrifugation at 4 °C. Total RNA was extracted using an RNA extraction kit (Tiangen Biotech, Beijing, China). Genomic DNA was removed using the gDNA wiper mix, and first-strand complementary DNA (cDNA) was synthesized using the HiScript II qRT Supermix (Vazyme Biotech, Nanjing, China) according to the manufacture’s recommendations. The expression of genes was analyzed using quantitative real-time PCR (RT-qPCR). The gene *rplT* was used as the reference gene and the primers used are presented in Supplementary Table S1. The fold-changes of these genes were normalized as described previously [28].

### 3.10. Statistical Analysis

All experiments were performed in duplicate. The groups were compared using one-way analysis of variance using the statistical software SPSS version 18.0. *p* values of ≤0.05 indicated significant differences between groups.

## 4. Discussion

Phloretin is a phenolic compound that is abundant in the skin and root bark of many kinds of juicy fruits such as apples, pears, and loquat [29]. Previous studies have shown that phloretin could inhibit the biofilm formation and virulence of *Streptococcus mutans* and *Escherichia coli* [30,31]. However, whether phloretin can inhibit the biofilm formation and pathogenicity of *S. marcescens* has not been documented. Here, the antivirulence potential of pholretin in combination with traditional antibiotics against *S. marcescens* NJ01 was evaluated, and the underlying mechanism was elucidated.

The formation of biofilms is among the most important factors leading to resistance in *S. marcescens* [32]. QS has been proven to play a crucial role in the mediation of biofilm formation [9]. Therefore, disrupting the QS of *S. marcescens* is a compelling method for controlling the drug resistance of this spoilage bacterium. *S. marcescens* can secrete a series of AHLs with chain lengths ranging from C4 to C8. Once AHLs accumulate to a certain level, they bind to their native receptors, thereby regulating the synthesis of biofilms and virulence factors [33]. In the present study, C4-HSL and C6-HSL were detected as the main AHLs secreted by *S. marcescens* NJ01. After exposure to pholoretin, the secretion of C4-HSL and C6-HSL was significantly reduced. The results indicated that pholoretin has a powerful suppressive effect against the QS. As biofilm formation was QS-mediated, the suppressed QS of *S. marcescens* would inevitably result in the reduced biomass of biofilms. The quantitative analysis indicated that the biofilms of *S. marcescens* were considerably diminished after exposure to phloretin. This result was consistent with Ramanathan et al., who reported that the disruption of QS inhibits the biofilm formation of *S. marcescens* [12]. Furthermore, RT-qPCR analysis indicated that the expression of *bsmA*, which is involved in biofilm development, was considerably repressed. The repressed expression of *bsmA* was consistent with the inhibited biofilm formation.

Notably, the phloretin-treated samples showed flat and scattered biofilms. The thick matrix wrapped on the surface of the biofilms was significantly reduced. Thus, the structure of the biofilms was disrupted. QS-mediated EPSs are crucial components of biofilms and play a critical role in blocking the access of antibiotics to cells [8]; therefore, we determined the impact of phloretin on EPS production. Our results indicated that EPS production was notably reduced after phloretin was administered. The reduced EPS production was correlated well with the RT-qPCR analysis results, in which the expression of *ebp*, a gene involved in EPS synthesis, was found to be downregulated [34].

Metabolomics research indicated that the level of ethanolamine was considerably increased after exposure to phloretin. Ethanolamine is a crucial ingredient of the cell membrane and takes the lead in sustaining membrane permeability [35]. The increased ethanolamine suggested that the membrane permeability might be changed. To validate our hypothesis, the permeability of membranes treated with phloretin was investigated. Our results indicated that phloretin considerably improved the permeability of the membrane. The altered biofilm structure and improved membrane permeability helped antibiotics enter the biofilm cells, eventually increasing the susceptibility of biofilm cells to mikacin, netilmicin, and imipenem. The improved efficiency of phloretin and antibiotics was similar to our previous study, which showed the notably enhanced susceptibility of *S. marcescens* toward ciprofloxacin [3].

Phloretin treatment also resulted in the significantly suppressed production of virulence factors including protease, prodigiosin, and swimming and swarming motilities. Protease secreted by *S. marcescens* is the main factor causing the spoilage and deterioration of dairy products [36,37]. Siddiqui et al. reported that *htpX* is involved in the synthesis of protease [38]. Here, the expression of *htpX* was considerably repressed after exposure to phloretin, which was consistent with the reduced protease activity. Prodigiosin is among the most typical virulence factors produced by *S. marcescens* and plays a vital role in host infection [39]. The synthesis of prodigiosin is controlled by *pig* gene clusters (*pigA*-*O*) [40]. Here, the expression of *pigC* and *pigM* was considerably downregulated after phloretin exposure. The repressed expression of *pigC* and *pigM* correlated well with the decreased prodigiosin level. Furthermore, the motilities of *S. marcescens* were considerably repressed after exposure to phloretin. Swimming and swarming motilities are essential for the attachment and development of *S. marcescens* biofilms [3]. The inhibition of motilities would result in reduced biofilm formation and pathogenicity. This speculation was validated by the results observed, in which biofilm formation was inhibited.

Glutamate is an important component of glutathione and plays an essential role in countering oxidative injury [41]. Betaine is essential for defending against oxidative stress and maintaining the integrity and normal function of the cell membrane [24]. Here, the levels of glutamate and betaine were considerably decreased after treatment with phloretin. Oxidative stress might be intensified, as glutamate and betaine levels were reduced. To defend against the intensified oxidative stress induced by phloretin and repair the damaged membrane, glutamate and betaine were excessively consumed. To confirm the oxidative injury caused by phloretin, the production of ROS and H_2_O_2_ was investigated. The data indicated that the production of ROS and H_2_O_2_ was considerably increased after phloretin treatment. A previous study showed that QS would improve the activity of antioxidant enzymes [42]. Here, the repressed expression of *katG* and *gpx*, which are involved in the synthesis of catalase and glutathione peroxidase, respectively, further confirmed that the QS of *S. marcescens* NJ01 was dysfunctional. Isoleucine and leucine are branched-chain amino acids and take the lead in the synthesis of functional proteins [43]. The notable reduction in isoleucine and leucine indicated that amino acid metabolism was disrupted due to oxidative stress and resulted in the attenuated virulence of *S. marcescens*, consistent with the reduced virulence factors.

Succinate and fumarate are important intermediate metabolites of the TCA cycle. The decrease in succinate and fumarate indicated that energy metabolism was notably disrupted. The disruption of the TCA cycle, as the most important energy source for micro-organisms, will inevitably lead to a shortage of the energy supply and the weakened pathogenicity of *S. marcescens* [24]. NAD^+^ is an important metabolite involved in nucleic acid metabolism. It also takes the lead in antioxidation and improving the survival rate of cells in stressful situations [44]. The reduced NAD^+^ indicated that the nucleic acid metabolism was disrupted, and also functioned as a cell self-repairing mechanism to resist oxidative injury.

## 5. Conclusions

In this study, we investigated the antivirulence capacity of phloretin against *S. marcescens* NJ01 and uncovered the underlying mechanism. Phloretin significantly inhibited the secretion of C4-HSL and C6-HSL, thus disrupting the QS system of this bacterium. Dysfunctional QS resulted in the suppressed formation of biofilms and production of virulence factors, as well as intensified oxidative injury. The improved injury altered components of the cell membrane, improved membrane permeability, and eventually enhanced the susceptibility of biofilm cells to antibiotics. Oxidative stress also resulted in disorders of amino acid metabolism, energy supply, and nucleic acid metabolism, eventually attenuating the pathogenicity of *S. marcescens*. Therefore, phloretin is expected to become an effective method for controlling vegetable spoilage induced by *S. marcescens*.

## Figures and Tables

**Table 1:** Metabolite assignment in *S. marcescens* NJ01.

No.	Compound	Assignments	Chemical Shift (ppm) ^a^	Fold ^b^	*p* ^c^
1	Glycocholate	CH3	0.93 (t)	0.1	
2	Isoleucine	d-CH3, g-CH3, a-CH	0.94 (t), 1.01 (d), 3.67 (d)	0.73	*
3	Leucine	d-CH3, d-CH3, a-CH	0.96 (t), 0.97 (d), 3.72 (d)	0.56	***
4	Valine	g-CH3, g-CH3, a-CH	0.99 (d), 1.05 (d), 3.59 (d)	0.07	
5	3-Methyl-2-oxovalerate	CH3	1.1 (d)	−0.67	
6	Lactate	CH3	1.33 (d)	1.13	***
7	Alanine	CH3, CH	1.49 (d), 3.78 (q)	0.92	***
8	Acetate	CH3	1.92 (s)	−0.07	
9	2-Aminoadipate	CH2	2.07 (m)	−1.05	*
10	Glutamate	b-CH2, g-CH2, a-CH	2.06 (m), 2.35 (dt), 3.76 (q)	−1.21	***
11	Succinate	CH2	2.41 (s)	−0.43	*
12	2-Oxoglutarate	CH	3.0 (t)	−0.96	
13	Ethanolamine	CH2	3.13 (t), 3.81 (t)	0.74	*
14	Betaine	CH2	3.23 (s)	−3.82	**
15	Glycine	CH2	3.56 (s)	0.7	***
16	Maltose	CH	3.63 (dd)	0.97	***
17	Uracil	CH	5.81 (d), 7.54 (d)	0.71	***
18	Fumarate	CH	6.52 (s)	−2.19	***
19	Tyrosine	2-CH, 6-CH	6.91 (d)	−0.52	**
20	Phenylalanine	ph-H	7.3–7.46 (m)	−0.3	**
21	Hypoxanthine	2-H, 8-H	8.2 (s), 8.22 (s)	0.03	
22	NAD^+^	7-CH, 39-CH	8.13 (s), 8.83 (s), 8.84 (d)	−0.86	*
23	Nicotinate	CH	8.89 (dd)	0.38	

^a^ Multiplicity: (s) singlet, (d) doublet, (t) triplet, (q) quartets, (m) multiplets. ^b^ Color-coded according to the log_2_(fold): red and blue represent the increased and decreased metabolites, respectively, in Ses-treated group. ^c^ *p* values were calculated based on a parametric Student *t* test or a nonparametric Mann–Whitney test and were corrected by the BH (Benjamini–Hochberg) methods; values with asterisk symbols denoted extent of significance: * *p* < 0.05, ** *p* < 0.01, and *** *p* < 0.001.

## Data Availability

All data generated or analyzed during this study are included in this published article.

## Supplementary Materials

The following supporting information can be downloaded at: https://www.mdpi.com/article/10.3390/molecules28248067/s1, Table S1: PCR primers for qRT-PCR.
