# Supplementary material for: Phloretin Inhibits Quorum Sensing and Biofilm Formation in Serratia marcescens
Source: Molecules. 2023 Dec 13;28(24):8067. doi: 10.3390/molecules28248067 (PMC10746122; doi:10.3390/molecules28248067)
Supplement: Supplementary file 1 [file molecules-28-08067-s001.zip › molecules-2706138-supplementary.pdf]

Article

Phloretin Inhibits Quorum Sensing and Biofilm Formation in *Serratia marcescens*

Yueheng Qi [1,2,†], Pengcheng Ji [3,†], Kunyuan Yin [3], Yi Zheng [3], Jiangxiu Niu [2], Aiqun Jia [1], Jinwei Zhou [3,*]
and Jingguo Li [1,*]

1 Henan Provincial People's Hospital, People's Hospital of Zhengzhou University, Zhengzhou 477150, China;
2 Luoyang Key Laboratory of Organic Functional Molecules, College of Food and Drug,
Luoyang Normal University, Luoyang 471934, China;
3 School of Food and Biological Engineering, Xuzhou University of Technology, Xuzhou 221018, China;
* Correspondence: zhoujinwei@xzit.edu.cn (J.Z.); lijingguo@zzu.edu.cn (J.L.); Tel.: +86-516-83105383 (J.Z.); +86-371-65896979 (J.L.); Fax: +86-516-83105383 (J.Z.)
† These authors contributed equally to this work.

Table S1 PCR primers for qRT-PCR.

Genes	Primer direction	Sequence (5′–3′)
katG	F	GACTTCTTCCGCCACCTGTTCG
	R	CGATGTCTTTCGCCACCCACTG
bsmA	F	TAGTCCGCACACTCATCGC
	R	GATCTCCTGCGCCTGTGC
bsmB	F	GCGGATGTGTATGCCTTCG
	R	GCCACGCATTTCTTCACTCA
fimC	F	CCAAATCCACGCCGGAAAAC
	R	TTGTTCTTGTCGCTGTCCGT
ebp	F	CTCAGAGGCACACTGGGCATTG
	R	ATTGTCAGCACCGATGGCGATC
pigM	F	GTTCGCTCAGTATCGCAGGTGAC
	R	GGTAGGCTGCCGTTCGTTGAAG
pigC	F	TTCGTCACAAACCGCACTATT
	R	CGTCTTTCACCGCCCATT
htpX	F	GCATCCTGGCGAGCATCATCAC
	R	GCAAGGCGGCGATCATCTTCTC
rplT	F	ATGAAGCAGGCGAAAGGTTACTACG
	R	CGACGGTCACGGTAAGCATACTG
